# Enoxaparin is associated with lower rates of mortality than unfractionated Heparin in hospitalized COVID-19 patients

**DOI:** 10.1016/j.eclinm.2021.100774

**Published:** 2021-03-09

**Authors:** Colin Pawlowski, AJ Venkatakrishnan, Christian Kirkup, Gabriela Berner, Arjun Puranik, John C. O'Horo, Andrew D. Badley, Venky Soundararajan

**Affiliations:** a nference inc., One Main Street, Suite 400, East Arcade, Cambridge, MA 02142, USA; b Mayo Clinic, Rochester, MN, USA

## Abstract

**Background:**

Coagulopathies are a major class among COVID-19 associated complications. Although anticoagulants such as unfractionated Heparin and Enoxaparin are both being used for therapeutic mitigation of COVID associated coagulopathy (CAC), differences in their clinical outcomes remain to be investigated.

**Methods:**

We analyzed records of 1,113 patients in the Mayo Clinic Electronic Health Record (EHR) database who were admitted to the hospital for COVID-19 between April 4, 2020 and August 31, 2020, including 19 different Mayo Clinic sites in Arizona, Florida, Minnesota, and Wisconsin. Among this patient population, we compared cohorts of patients who received different types of anticoagulants, including 441 patients who received unfractionated Heparin and 166 patients who received Enoxaparin. Clinical outcomes at 28 days were compared, and propensity score matching was used to control for potential confounding variables including: demographics, comorbidities, ICU status, chronic kidney disease stage, and oxygenation status. Patients with a history of acute kidney injury and patients who received multiple types of anticoagulants were excluded from the study.

**Findings:**

We find that COVID-19 patients administered unfractionated Heparin but not Enoxaparin have higher rates of 28-day mortality (risk ratio: 4.3; 95% Confidence Interval [C.I.].: [1.8, 10.2]; *p*-value: 8.5e−4, Benjamini Hochberg [BH] adjusted *p*-value: 2.1e−3), after controlling for potential confounding factors.

**Interpretation:**

This study emphasizes the need for mechanistically investigating differential modulation of the COVID-associated coagulation cascades by Enoxaparin versus unfractionated Heparin.

**Funding:**

This work was supported by Nference, inc.

**Research in Context**

**Evidence before this study**

Prior studies have suggested that Enoxaparin significantly reduces venous thromboembolism compared with unfractionated Heparin, and is also associated with reduced all-cause mortality. In addition, coagulopathies are among the major complications of COVID-19, and as a result anticoagulants have become a standard course of treatment for this indication. However, thus far there have been few studies comparing the efficacy of different anticoagulant treatments in the context of COVID-19.

**Added value of this study**

This study provides a comparison of Enoxaparin vs. unfractionated Heparin for the treatment of hospitalized patients with COVID-19. We find that COVID-19 patients administered unfractionated Heparin but not Enoxaparin have higher rates of 28-day mortality, even after controlling for potential confounding factors.

**Implications of all the available evidence**

This findings from this retrospective study motivates prospective clinical studies to investigate the differences in the patient outcomes associated with administration of different types of anticoagulants in hospitalized COVID-19 patients. While the findings suggest that patients administered Enoxaparin fare better, this may reflect different therapeutic and prophylactic treatment patterns of the two medications. Further pre-clinical and clinical investigations to understand the mechanistic differences in efficacy for these anticoagulants and other agents also warranted.

Alt-text: Unlabelled box

## 1 Introduction

COVID-19 manifests in varying levels of patient outcomes ranging from mild, moderate to severe disease [1], [2], [3]. While the mild/asymptomatic patients have been managed through quarantining and self-medication, the optimal management of hospitalized moderately ill or severely ill COVID-19 patients remains a formidable challenge. Owing to the diversity of comorbidities and complications in the afflicted patients and the overwhelming pace of the pandemic, the regimen of medications in COVID-19 critical care is yet to be standardized. Meanwhile, the accumulation of real-world data on patient outcomes of COVID-19 from healthcare systems provides an excellent opportunity to identify underlying trends that could lead to actionable insights.

Coagulopathies are a major class among COVID-19 associated complications [4], particularly in a critical care setting [5]. Prior studies have provided a fine-grained resolution of the hematological parameters in COVID-19 patients [6]. Trials comparing anticoagulation treatments are ongoing, however owing to the wide-spread and severe impact of the disease, there is a need for evaluation of observational data in order to rapidly generate evidence to guide treatment [7]. A spectrum of anticoagulants such as unfractionated Heparin, Enoxaparin, and Rivaroxaban are being used in COVID-19 patient management as needed [8,9]. A clinical trial is being designed to examine whether prophylactic-dose Enoxaparin improves survival and reduces hospitalizations in older (age > 50) symptomatic ambulatory patients [10]. Recent in vitro studies have shown that pseudotyped viral particles were efficiently neutralized by a variety of anticoagulants [11]. In addition, results from a phase II clinical trial found that patients treated with therapeutic Enoxaparin had improved gas exchange and more ventilator-free days compared to patients treated with standard anticoagulant thromboprophylaxis [12]. Furthermore, previous studies and meta-analyses have suggested that Enoxaparin may be more efficacious than unfractionated Heparin in preventing VTE [13]. In this study, we focused on Enoxaparin and unfractionated Heparin because these are among the frequently used anticoagulant agents, and prior studies have been performed doing a head-to-head comparison of these two medications across a variety of indications [14], [15], [16].

Based upon the findings from these previous studies, we hypothesize that hospitalized COVID-19 patients administered Enoxaparin may have better clinical outcomes compared to patients administered unfractionated Heparin. The availability of clinical covariates and outcomes of patients administered these medications at Mayo Clinic sites and associated health systems enables us to evaluate this hypothesis. Here, we present a comparison of the patient outcomes in terms of mortality status, ICU admission and the durations of stay in ICU and hospital in severe COVID-19 patients that were administered unfractionated Heparin vs. Enoxaparin. We use propensity score matching to construct matched cohorts for comparison which are balanced across a range of clinical covariates, including: demographics, comorbidities, admission diagnosis, initial ICU status, and initial level of oxygen support. More details on the study design, propensity score matching, curation of clinical covariates, and statistical analyses are provided in the *Methods* section. We summarize the findings from the study and discuss the results in the *Results* and *Discussion* sections, respectively.

## 2 Methods

### 2.1 Institutional review board (IRB)

This retrospective research was conducted under IRB 20–003278, ‘Study of COVID-19 patient characteristics with augmented curation of Electronic Health Records (EHR) to inform strategic and operational decisions’. Under this IRB, all the authors had access to the EHR records for all Mayo Clinic patients that were tested using a PCR based method for SARS-CoV-2. VS, ADB and JCO had access March 2020 onwards; CP had access May 2020 onwards; AJV, CK and GB had access July 2020 onwards, and AP had access October 2020 onwards. The study was deemed exempt by the Mayo Clinic institutional review board and waived from consent. Subjects without research authorization on file were excluded. For further information regarding the Mayo Clinic Institutional Review Board (IRB) policy, and its institutional commitment, membership requirements, review of research, informed consent, recruitment, vulnerable population protection, biologics, and confidentiality policy, please refer to www.mayo.edu/research/institutional-review-board/overview.

### 2.2 Study design

For this observational study, we considered all patients who were admitted to one of the hospitals in the Mayo Clinic system between April 5, 2020 and August 31, 2020, with a positive SARS-CoV-2 PCR test within 7 days prior to admission or during admission to the hospital. This included 1113 patients admitted to 19 Mayo Clinic sites in Arizona, Florida, Minnesota, and Wisconsin. The EHR data for this study was accessed between October 1, 2020 and November 5, 2020, and all of the authors had access to the underlying EHR data. We note that all data that was used for this study was extracted from a single EHR system, so no data linkage was required. Data available for analysis included patient demographics (age, gender, race, etc.), structured clinical data (medications administered, vital signs, ICD-10 diagnoses, etc.), and unstructured clinical data (physician notes). Using this information, a dataset was assembled including key covariates and outcomes measures derived from structured and unstructured clinical data. Among this patient population, 24 patients were excluded from the analysis based upon missing demographic information (age, gender, race, or ethnicity).

From this EHR dataset with 1089 patients with demographic information available, two cohorts were constructed: (i) patients who were administered Enoxaparin but not unfractionated Heparin within 28 days of their hospital admission for COVID-19 and (ii) patients who were administered unfractionated Heparin but not Enoxaparin within 28 days of their hospital admission for COVID-19. The medication codes used to identify orders for Enoxaparin and unfractionated Heparin are provided in **Supplementary Table S1**. In order to avoid potential confounding with underlying kidney conditions, patients with admission diagnoses or complications of acute kidney injury were excluded from this analysis. The ICD-10 codes used to identify patients with acute kidney injury are provided in **Supplementary Table S2**. The final cohort sizes were 441 and 166 for the Enoxaparin and unfractionated Heparin cohorts, respectively. The clinical characteristics of the two cohorts are provided in Table 1.

**Table 1:** Summary of patient characteristics for matched and original cohorts of hospitalized COVID-19 patients who have taken either Heparin or Enoxaparin. For numeric variables such as age, the mean value for each cohort is shown with standard deviation in parentheses. For categorical variables such as race and ethnicity, patient counts are shown with the percentage of each cohort in parentheses.

Clinical covariate	Enoxaparin, but not Heparin (Matched)	Heparin, but not Enoxaparin (Matched)	Enoxaparin, but not Heparin (Original)	Heparin, but not Enoxaparin (Original)
Total number of patients	96	96	441	166
Age in years (standard deviation)	61.9 (17.5)	60.1 (19.6)	57.1 (17.4)	62.0 (18.7)
Sex - Female - Male	46 (48%) 50 (52%)	39 (41%) 57 (59%)	218 (49%) 223 (51%)	58 (35%) 108 (65%)
Race - Asian - Black - Other - White	4 (4.2%) 7 (7.3%) 20 (21%) 65 (68%)	5 (5.2%) 7 (7.3%) 22 (23%) 62 (65%)	30 (6.8%) 49 (11%) 83 (19%) 279 (63%)	6 (3.6%) 15 (9%) 36 (22%) 109 (66%)
Ethnicity - Hispanic	20 (21%)	21 (22%)	92 (21%)	29 (17%)
Comorbidities in year prior to COVID-19 hospital admission - Cancer - Cardiac arrhythmia - Chronic kidney disease (based on physician notes) - Chronic pulmonary disease - Dementia - Depression - Diabetes - Hypertension - Hypothyroidism - Obesity - Stroke/neurologic disorders	6 (6.2%) 5 (5.2%) 14 (15%) 10 (10%) 4 (4.2%) 9 (9.4%) 20 (21%) 5 (5.2%) 11 (11%) 15 (16%) 5 (5.2%)	4 (4.2%) 2 (2.1%) 11 (11%) 8 (8.3%) 2 (2.1%) 5 (5.2%) 18 (19%) 4 (4.2%) 8 (8.3%) 17 (18%) 5 (5.2%)	25 (5.7%) 19 (4.3%) 21 (4.8%) 37 (8.4%) 8 (1.8%) 24 (5.4%) 64 (15%) 10 (2.3%) 35 (7.9%) 60 (14%) 13 (2.9%)	11 (6.6%) 11 (6.6%) 47 (28%) 18 (11%) 4 (2.4%) 8 (4.8%) 45 (27%) 9 (5.4%) 20 (12%) 25 (15%) 13 (7.8%)
CKD stage based on eGFR median value over prior year - Stage 3a - Stage 3b - Stage 4 - Stage 5	9 (9.4%) 1 (1%) 0 (0%) 0 (0%)	9 (9.4%) 1 (1%) 0 (0%) 0 (0%)	25 (5.7%) 3 (0.68%) 1 (0.23%) 0 (0%)	18 (11%) 25 (15%) 20 (12%) 0 (0%)
ICU admission on first day of hospitalization	6 (6.2%)	8 (8.3%)	19 (4.3%)	15 (9%)
First day of anticoagulation in ICU	14 (15%)	15 (16%)	56 (13%)	32 (19%)
Admission diagnosis - Bacterial pneumonia - Cardiac arrhythmias - Delirium / Encephalopathy - Sepsis - Stroke	0 (0%) 2 (2.1%) 2 (2.1%) 7 (7.3%) 1 (1%)	0 (0%) 1 (1%) 1 (1%) 6 (6.2%) 2 (2.1%)	2 (0.45%) 5 (1.1%) 10 (2.3%) 18 (4.1%) 1 (0.23%)	0 (0%) 4 (2.4%) 4 (2.4%) 12 (7.2%) 4 (2.4%)
Oxygenation on day of admission - Invasive mechanical ventilation - Non-invasive mechanical ventilation - High-flow oxygen therapy - Low-flow oxygen therapy - Other form of oxygen therapy - No oxygenation	2 (2.1%) 1 (1%) 5 (5.2%) 26 (27%) 0 (0%) 67 (70%)	2 (2.1%) 2 (2.1%) 7 (7.3%) 29 (30%) 0 (0%) 63 (66%)	3 (0.68%) 12 (2.7%) 33 (7.5%) 191 (43%) 2 (0.45%) 233 (53%)	15 (9%) 5 (3%) 8 (4.8%) 49 (30%) 1 (0.6%) 96 (58%)
Propensity score for Heparin vs. Enoxaparin treatment (standard deviation)	0.526 (0.183)	0.530 (0.185)	0.342 (0.184)	0.658 (0.27)

In order to account for potentially confounding variables, we performed propensity score matching to balance covariates between the two cohorts. The statistical tests for differences in outcomes were repeated on the matched cohorts. The covariates which were balanced include demographics, comorbidities, admission diagnoses, and other clinical covariates recorded upon admission. Admission diagnoses were defined as diagnoses recorded on the day of admission to the hospital, or within 7 day prior to admission. Comorbidities were defined as diagnoses recorded 7 days to one year prior to admission to the hospital. The ICD-10 codes used to define the admission diagnoses and comorbidities of interest are provided in **Supplementary Table S2**. More details on the propensity score matching procedure are provided in a subsequent section.

To compare these two matched cohorts, statistical tests were applied to a number of outcomes, including: (1) mortality status (i.e. was the patient ever recorded as deceased), (2) 28-day confirmed mortality status (i.e. of patients for whom we have some confirmation of mortality status at 28 days following hospitalization, was the patient among the deceased), (3) ICU admission during hospitalization, (4) length of stay in the hospital (among alive patients), (5) length of stay in ICU (among alive patients). Complications derived from ICD-10 codes were also considered, including: bacterial pneumonia, cardiac arrest, cardiac arrhythmia, co- or secondary infection, liver dysfunction, pleural effusions, pulmonary embolism, stroke/cerebrovascular incident, and viral pneumonitis. Complications were defined as diagnoses recorded within 28 days of their hospital admission for COVID-19. ICD-10 codes used to define the complications of interest are provided in **Supplementary Table S2**. In addition, neural network models were applied to identify thrombotic events and bleeding complications from the physician notes, including: bleeding, disseminated intravascular coagulation, hematemesis, hematoma, myocardial infarction, pulmonary embolism, purpura, stroke / cerebrovascular incident, and venous thromboembolism / deep vein thrombosis. More details on the neural network models used to identify the phenotypes from the physician notes are provided in a subsequent section.

The total population data used for this study was extracted from the EHR system and went through a minimal cleaning process to organize the data into relevant domains (e.g. Demographics, medications, Admission Discharge transfer, etc.) with standardized codes such as ICD-10 codes for diagnoses. The Pandas software package in Python was used to transform the data in preparation for the statistical analyses for this study.

### 2.3 Statistical analysis

Risk Ratios for Admission to the ICU and Mortality Status were calculated for patient cohorts defined by anticoagulant usage and presence of select comorbidities. Risk Ratio across two cohorts of interest is calculated by dividing the proportion of cohort 1 which responds affirmatively to a feature (or all members of a set of features) by the proportion of cohort 2 which responds affirmatively to the same feature (or set of features). Risk Ratio along with accompanying 95% confidence intervals for each cohort pair are calculated. Confidence intervals were computed using a Delta method approach [17].

A Pearson's Chi-Square test was run to test for statistically significant differences in proportion of occurrence of features across cohort pairs. This was done using the stats.chi2_contingency function of the scipy python package. Test statistics and *p*-values are reported for each cohort pair.

A two-sided Mann-Whitney test was performed to identify statistically significant differences in Hospital Length of Stay and ICU Length of Stay across two cohorts of patients who were discharged from the hospital alive and received different anticoagulants (unfractionated Heparin and Enoxaparin). P-values are reported for these tests. This test was implemented using the software package scipy in Python.

As we are comparing multiple outcomes between two anticoagulant cohorts, a Benjamini-Hochberg correction is applied to a set of outcomes. Mortality was identified as a primary outcome prior to performing the tests, so it may not be necessary to apply the Benjamini-Hochberg correction on that *p*-value; however, adjusted *p*-values are reported for all outcomes.

### 2.4 Propensity score matching

In order to control for potential confounding factors, propensity score matching [18] was performed. The two cohorts that were balanced were (i) the 441 patients (without acute kidney injury) who were administered Enoxaparin but not unfractionated Heparin and (ii) the 166 patients (without acute kidney injury) who were administered unfractionated Heparin but not Enoxaparin. Propensity scores were computed by fitting an l-1 regularized logistic regression model to predict which of the 2 cohorts the patient was in, as a function of the covariates (listed further below). The software package sklearn v0.20.3 in Python was used to train the l-1 regularized logistic regression model. After computing propensity scores, 1:1 matching was done, using a heuristic caliper of 0.1 times the pooled standard deviation and allowing for drops [19]. 104 matched pairs were found. After checking for quality of cohort balance (see Table 1), the same statistical procedures (chi-square test, Mann-Whitney test, risk ratio) were then run on the two matched cohorts of 104 patients each to identify if differences in outcome persist after the adjustments.

The covariates used for balancing are the following. Note that all variables below except for age and eGFR median value over the prior year are binary. In particular, categorical variables such as race and ethnicity are split into multiple dummy variables for the balancing step: *age (in years); gender:* female, male; *race:* white, asian, black, other; *Ethnicity:* Non-Hispanic, Hispanic, Other; *Comorbidities:* whether or not the patient has each of the following comorbidities, (1) cancer, (2) cardiac arrhythmias, (3) chronic kidney disease, (4) chronic pulmonary disease, (5) dementia, (6) depression, (7) diabetes, (8) hypertension, (9) obesity, (10) stroke or other neurological disorders; *eGFR median value over prior year*: in order to account for possible confounding due to underlying difference in kidney function between the cohorts, median eGFR value over tests taken in the year prior to hospital admission was computed and used as a covariate for balancing; *In ICU on admission to hospital; In ICU on first day of anticoagulant administration*: whether the patient was in the ICU on the first day they received the anticoagulant; *Diagnosis on admission:* whether or not the patient is reported to have each of the following conditions on admission to hospital. (1 bacterial pneumonia, (2) cardiac arrhythmias, (3) delirium / encephalopathy, (4) sepsis, (5) stroke; *Oxygenation status on day of admission:* whether or not the patient is reported to have received the following forms of oxygen therapy on admission.

For the above covariates, the only variable with missing data was ethnicity (98% complete). This variable was completed using median imputation prior to the propensity score matching step.

### 2.5 Inverse probability of treatment weighting

In addition to propensity score matching, we performed an inverse probability of treatment weighting (IPTW) analysis to adjust for the same set of covariates [20]. The IPTW weight was derived by first computing propensity scores, and then, for each patient with propensity score p: if the patient is in the treatment-positive cohort, the IPTW weight is 1/p, and if the patient is in the treatment-negative cohort, the IPTW weight is 1/(1-p).

Relative risk confidence intervals and p-values were computed using a bootstrap approach [21]. In particular, 500 bootstrap resamples were done. Full propensity score computation, and then IPTW procedure was done for each resample. Standard error of log(relative risk) was estimated as the standard deviation of the 500 log(relative risk) values derived from the bootstrap samples. Both 95% confidence intervals and p-values were then computed based on this standard error against the point estimate of the relative risk. [21] The relative risk proportions were smoothed to avoid zero denominators.

### 2.6 Kaplan-Meier survival curves and Cox proportional hazard model

**Fig. 1:**
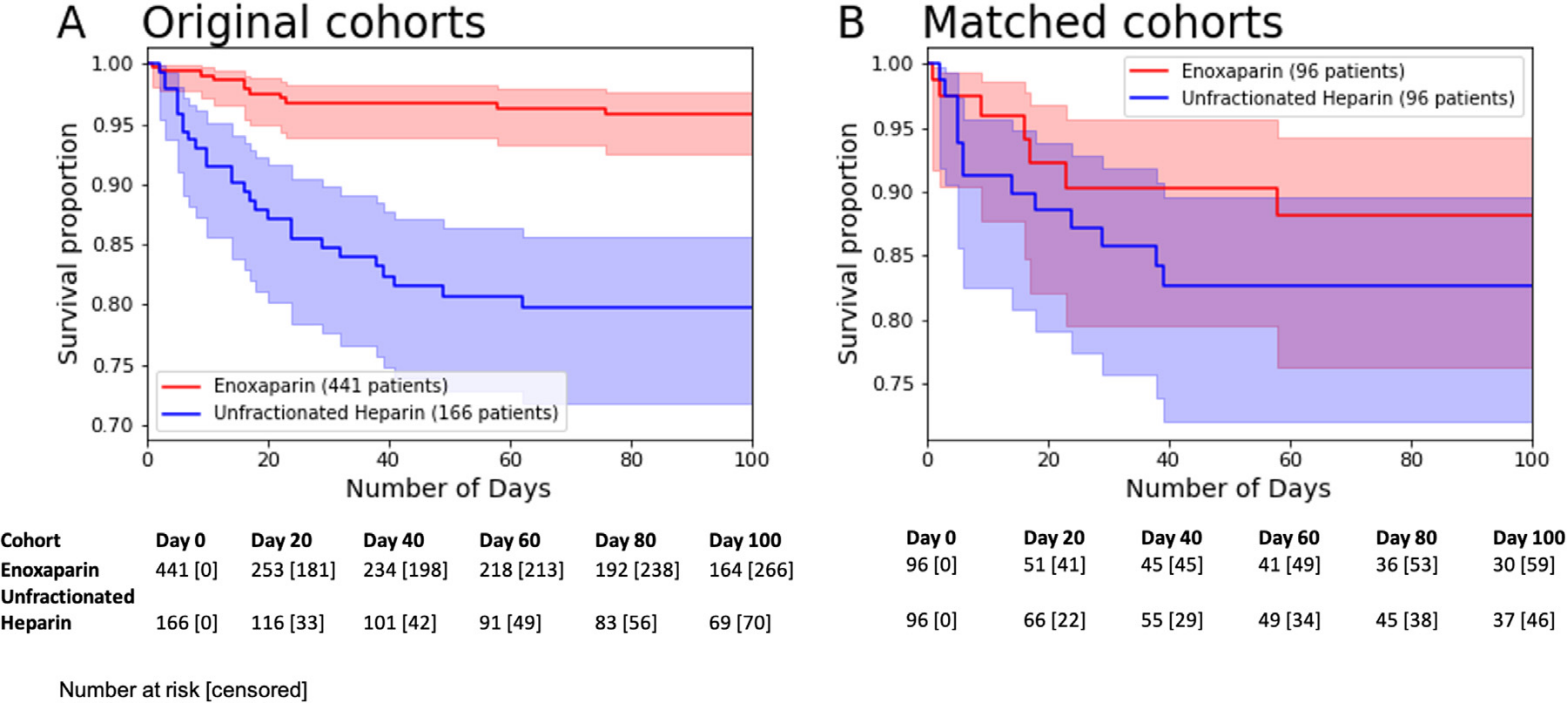
Kaplan-Meier survival curves for original and matched unfractionated Heparin and Enoxaparin cohorts. The total number of at risk patients are shown below for each time point, along with the number of censored patients in brackets.

We conducted some additional statistical analyses to assess the impact of anticoagulant treatment upon the mortality outcome variable. First, we plotted Kaplan-Meier survival curves to show the survival rate in the matched and unmatched cohorts over time (see Fig. 1), along with exponential Greenwood confidence intervals [22] as error bars. These plots were generated using the software package Lifelines v0.25.6 in Python.

In addition, we trained a Cox proportional hazard model [23] to estimate the relative impact of each of the clinical covariates upon survival, while holding all of the other clinical covariates constant. For the categorical variables with the multiple options, a single option is selected as the “reference”, which by definition has a hazard ratio of 1.0. The clinical covariates for this model and the reference categories include – *Anticoagulant administered:* Unfractionated Heparin (reference), Enoxaparin; *Age (in years); Gender:* Female (reference), Male. *Race:* White (reference), Asian, Black, Other. *Ethnicity:* Non-Hispanic (reference), Hispanic, Other. *Oxygenation status on hospital admission:* None (reference), Invasive mechanical ventilation, Low-flow oxygen, Non-invasive mechanical ventilation, Other. *Chronic kidney disease staging:* None (reference), Stage 3a (eGFR: 45–59%), Stage 3b (eGFR: 30–44%), Stage 4 (eGFR: 15–29%). *Comorbidity:* For each comorbidity, the reference category was considered to be the absence of the comorbidity: Cancer, Cardiac arrhythmias, Chronic pulmonary disease, Dementia, Depression, Hypertension, Hypothyroidism, Obesity, Pulmonary embolism, Stroke or other neurological disorders, Type 1 diabetes mellitus, Type 2 diabetes mellitus; *Admitted to the ICU on first day of hospital admission:* False (reference), True; *Admitted to the ICU on first day of anticoagulant administration:* False (reference), True.

The Cox proportional hazard model was trained using the software package Lifelines v0.25.6 in Python, with a penalizer term of 0.01.

### 2.7 Stratified analysis by dosing type

In addition to the primary analyses comparing the unfractionated Heparin and Enoxaparin cohorts, we also considered the mortality outcomes for both cohorts receiving prophylactic and therapeutic doses of these anticoagulants. For unfractionated Heparin, administration frequency is used to determine the dosing type (therapeutic: continuous administration, prophylactic: periodic administration). For Enoxaparin, doses above 40 mg were considered to be therapeutic, and doses less than or equal to 40 mg are considered to be prophylactic [24].

### 2.8 Augmented curation of thrombotic events and bleeding phenotypes from the unstructured text of the electronic health record (EHR) clinical notes

We used a previously developed state-of-the-art Bidirectional Encoder Representations from Transformers (BERT)-based neural network to classify sentiment of clinical manifestations and diagnoses in the EHR [2]. Specifically, the model extracts sentences containing clinical phenotypes and classifies their sentiment into the following categories: Yes (confirmed clinical manifestation or diagnosis), No (ruled out clinical manifestation or diagnosis), Maybe (possibility of clinical manifestation or diagnosis), and Other (alternate context, e.g. family history of disease). The model was trained using 18,490 sentences and approximately 250 phenotypes with an emphasis on cardiovascular, pulmonary, and metabolic phenotypes. It achieves 93.6% overall accuracy and over 95% precision and recall for both “Yes” and “No” sentiment classification [2]. Furthermore, this model has also been applied and validated in a previous study to identify thrombotic events for patients with COVID-19^6^. We applied this neural network model to identify the following phenotypes from the physician notes: bleeding, disseminated intravascular coagulation, hematemesis, hematoma, myocardial infarction, pulmonary embolism, purpura, stroke / cerebrovascular incident, and venous thromboembolism / deep vein thrombosis.

### 2.9 Cause of death information

For the deceased patients in the matched Enoxaparin and Heparin cohorts, we conducted a manual review of the clinical notes to determine the cause of death. This manual review was carried out by the study authors CP, AV, CK, and GB. The potential causes of death that were considered included: (1) Acute respiratory distress syndrome / acute respiratory failure / hypoxia, (2) Acute kidney injury / renal failure, (3) Sepsis, (4) Pneumonia, (5) Heart failure, (6) Other (none of the causes 1–5), and (7) Inconclusive (cause of death could not be determined from the manual review). The results from this manual curation are presented in **Supplementary Table S3**.

**Role of the funding source:** The funder was involved in the design and conduct of the study and data management.

## 3 Results

In Fig. 1, we show Kaplan-Meier survival curves for the original and matched unfractionated Heparin and Enoxaparin cohorts. The outcomes for the original and matched cohorts are presented in Table 2 and Table 3, respectively. In the following sections, we provide a detailed description and interpretation of these results and secondary analyses.

**Table 2:** Summary of clinical outcomes for **unmatched** cohorts of hospitalized COVID-19 patients who have taken either Heparin or Enoxaparin. For categorical variables such as mortality status and complications, patient counts are shown with the percentage of each cohort in parentheses. For numeric variables such as hospital and ICU length of stay, the mean value for each cohort is shown with standard deviation in parentheses. In addition, Benjamini-Hochberg adjusted *p*-values are shown for the statistical tests comparing the outcome variables for the matched Enoxaparin and Heparin cohorts.

Outcome variable	Enoxaparin, but not Heparin (Original)	Heparin, but not Enoxaparin (Original)	*p*-value	BH-adjusted *p*-value	Risk ratio (95% CI)
Number of patients	441	166			
Deceased (ever)	11 (2.5%)	28 (17%)	2.7e−9	1.3e−8	6.76 (3.39, 12.7)
Deceased 28 days within first day of hospitalization (out of patients with known mortality status)	9/244 (3.7%)	20/118 (17%)	3.8e−5	4.7e−5	4.60 (2.13, 9.29)
ICU admission	88 (20%)	50 (30%)	9.1e−3	0.01	1.51 (1.12, 2.03)
Hospital length of stay in days	5.4 (4.3)	7.4 (6.6)	0.02	0.02	
ICU length of stay in days	0.92 (2.5)	2.3 (5.4)	4.5e−3	7.5e−3

**Table 3:** Summary of clinical outcomes for **matched** cohorts of hospitalized COVID-19 patients who have taken either Heparin or Enoxaparin. For categorical variables such as mortality status and complications, patient counts are shown with the percentage of each cohort in parentheses. For numeric variables such as hospital and ICU length of stay, the mean value for each cohort is shown with standard deviation in parentheses. In addition, Benjamini-Hochberg adjusted p-values are shown for the statistical tests comparing the outcome variables for the matched Enoxaparin and Heparin cohorts.

Outcome variable	Enoxaparin, but not Heparin (Matched)	Heparin, but not Enoxaparin (Matched)	*p*-value	BH-adjusted *p*-value	Risk ratio (95% CI)
Number of patients	96	96			
Deceased (ever)	7 (7.3%)	13 (13.5%)	0.24	0.59	1.86 (0.77, 4.20)
Deceased 28 days within first day of hospitalization (out of patients with known mortality status)	6/55 (11%)	10/61 (16%)	0.43	0.72	1.50 (0.59, 3.62)
ICU admission	21 (22%)	23 (24%)	0.86	0.86	1.09 (0.65, 1.83)
Hospital length of stay in days	5.6 (3.9)	5.6 (5.6)	0.17	0.59	
ICU length of stay in days	0.90 (2.0)	1.4 (4.2)	0.75	0.86	

### 3.1 Patients that were administered enoxaparin have lower mortality rates, lower ICU admission rates, and shorter hospital / ICU stays

We compared the mortality rate and ICU admission rate in patients from the curated Mayo Clinic dataset of 607 hospitalized COVID-19 patients (with no acute kidney injury) who received either Enoxaparin or Heparin, but not both. Of these patients, 441 were administered Enoxaparin but not unfractionated Heparin; of these 11 (2.5%) were deceased. For comparison, 166 patients were administered Heparin but not Enoxaparin. Of these patients, 28 (17%) were deceased. Comparing the mortality outcomes, patients in the Heparin only cohort have a higher mortality rate than those in the opposing Enoxaparin cohort (risk ratio of death: 6.76; 95% C.I.: [3.39, 12.7]; adjusted *p*-value <0.0001) (Table 2). Of the 441 patients administered Enoxaparin but not Heparin, 88 (20%) were later admitted to the ICU. Similarly, of the 166 patients administered Heparin but not Enoxaparin, 50 (30%) were admitted to the ICU. Comparing the ICU admission status, patients administered Heparin had a higher admission to ICU rate compared to Enoxaparin (risk ratio of ICU admission: 1.51; 95% C.I.: [1.12, 2.03]; adjusted *p*-value 0.01) (Table 2).

Next, we compared the lengths of stay in the ICU and the overall length of stay in the hospital. Here, we restricted the analysis to only patients that were alive. The length of stay in the ICU and in the hospital were shorter for patients administered Enoxaparin but not Heparin (mean ICU duration: 0.9 days [standard deviation: 2.5], mean hospital duration: 5.4 days [standard deviation: 4.3]) compared to the patients administered Heparin but not Enoxaparin (mean ICU duration: 2.3 days [standard deviation: 5.4], mean hospital duration: 7.4 days [standard deviation: 6.6]) (Table 2). The difference in hospital length of stay and ICU length of stay across the two cohorts are both statistically significant according to a *t*-test (hospital duration adjusted *p*-value: 0.02; ICU duration adjusted *p*-value: 7.5e−3). Taken together, this suggests preliminarily that Enoxaparin is correlated with a lower mortality rate, ICU admission rate and ICU and hospital length of stay compared to Heparin. Also of note are the increased rate of co-/secondary infection (5.4% vs 0.68%; adjusted *p*-value 7.1e−4) in the Heparin cohort (Table 4). On examination of cause of death for deceased patients for both cohorts, it is clear that the majority of deaths occurring in both the Heparin and Enoxaparin cohorts are driven primarily by COVID-19 associated causes, giving an indication that divergent rates of mortality between the cohorts is not the result of extraneous illness or accidents (**Supplementary Table S3**).

**Table 4:** Summary of occurrences of complications during hospitalization (days 0 to 28) for **unmatched** cohorts of hospitalized COVID-19 patients who have taken either Heparin or Enoxaparin.

Complication	Enoxaparin, but not Heparin (Original)	Heparin, but not Enoxaparin (Original)	Chi-square *p*-value	BH-adjusted *p*-value
Number of patients	441	166		
Bacterial pneumonia	3 (0.68%)	4 (2.4%)	0.09	0.51
Cardiac arrest	0 (0%)	0 (0%)	1.00	1.00
Cardiac arrhythmia	3 (0.68%)	1 (0.6%)	1.00	1.00
Co- or secondary infection	3 (0.68%)	9 (5.4%)	7.1e−4	0.01
Liver dysfunction	5 (1.1%)	3 (1.8%)	0.46	1.00
Pleural Effusions	0 (0%)	0 (0%)	1.00	1.00
Pulmonary embolism	3 (0.68%)	3 (1.8%)	0.35	0.97
Stroke / Cerebrovascular incidents	3 (0.68%)	2 (1.2%)	0.62	1.00
Viral pneumonitis	71 (16%)	21 (13%)	0.31	0.97

### 3.2 After controlling for potential confounding variables, patients that were administered enoxaparin have lower mortality rates

To simultaneously account for the effects of a range of possible confounders, we also examined statistical differences in outcomes between 1:1 propensity matched cohorts of patients who received Enoxaparin but not Heparin vs patients who received Heparin but not Enoxaparin. These cohorts were originally 441 and 166 patients, respectively; after 1:1 propensity score matching we were left with 2 cohorts of size 96; we refer to these cohorts as the “matched Enoxaparin” and “matched Heparin” cohorts respectively. Quality of balance between covariates is shown in Table 1. Most covariates (including demographics, comorbidities, and conditions on admission) are well-matched.

Of the 96 patients in the matched Enoxaparin cohort, 7 (7.3%) were eventually deceased; in the matched Heparin cohort, 13 (13.5%) were deceased (Table 3). The rate of mortality in the matched Heparin cohort was higher, though not statistically significant (adjusted Mann-Whitney *p*-value 0.59) (Table 3). The risk ratio of mortality for Heparin patients is 1.86 (95% CI: [0.77, 4.20]). Mean hospital length of stay (among alive patients) for matched Heparin was 5.6 days [standard deviation 5.6] vs 5.6 days [standard deviation 3.9] for matched Enoxaparin. Mean ICU length of stay (among alive patients) for matched Heparin was 1.4 days [standard deviation 4.2] vs 0.9 days [standard deviation 2.0] for matched Enoxaparin patients. Neither difference was significant (hospital duration adjusted p-value: 0.37; ICU duration adjusted *p*-value 0.25). Difference in rates of certain complications among the matched cohorts were analyzed. (Table 5). Counts were generally small, and no differences were statistically significant.

**Table 5:** Summary of occurrences of complications during hospitalization (days 0 to 28) for **matched** cohorts of hospitalized COVID-19 patients who have taken either Heparin or Enoxaparin.

Complication	Enoxaparin, but not Heparin (Original)	Heparin, but not Enoxaparin (Original)	Chi-square *p*-value	BH-adjusted p-value
Number of patients	96	96		
Bacterial pneumonia	0 (0%)	0 (0%)	1	1
Cardiac arrest	0 (0%)	0 (0%)	1	1
Cardiac arrhythmia	3 (3.1%)	1 (1.0%)	0.62	1
Co- or secondary infection	2 (2.1%)	2 (2.1%)	1	1
Liver dysfunction	2 (2.1%)	1 (1.0%)	1	1
Pleural Effusions	0 (0%)	0 (0%)	1	1
Pulmonary embolism	0 (0%)	1 (1.04167%)	1	1
Stroke / Cerebrovascular incidents	2 (2.1%)	0 (0%)	0.50	1
Viral pneumonitis	12 (13%)	9 (9.4%)	0.64	1

As an alternate approach to accounting for these confounders, inverse probability of treatment weighting (IPTW) was also done (using the same propensity scores as in matching to define weights). Covariate balance between the weighted cohorts is shown in Table 6. A significant difference in rate of mortality between the weighted Heparin and weighted Enoxaparin cohorts is observed; 14% of the weighted Heparin cohort were later deceased, while 2.7% of the weighted Enoxaparin cohort were later deceased, giving a risk ratio of mortality of 5.2 (bootstrap 95% CI [2.5, 10.7]; adjusted bootstrap *p*-value <0.0001) (Table 7). No significant differences between rates of ICU admission, ICU length of stay, or hospital length of stay (Table 7), or in rates of the various complications (Table 8).

**Table 6:** Summary of patient characteristics for **weighted** and original cohorts of hospitalized COVID-19 patients who have taken either Heparin or Enoxaparin. For numeric variables such as age, the mean value for each cohort is shown with standard deviation in parentheses. For weighted cohorts, proportions are computed as the total weight of patients who have the feature divided by the sum of weights over all patients.

Clinical covariate	Enoxaparin, but not Heparin (Matched)	Heparin, but not Enoxaparin (Matched)	Enoxaparin, but not Heparin (Original)	Heparin, but not Enoxaparin (Original)
Total number of patients	441	166	441	166
Age in years (standard deviation)	59.1 (17.5)	60.0 (19.5)	57.1 (17.4)	62.0 (18.7)
Sex - Female - Male	45% 55%	43% 57%	218 (49%) 223 (51%)	58 (35%) 108 (65%)
Race - Asian - Black - Other - White	6.2% 10% 20% 64%	5.6% 9.3% 20% 65%	30 (6.8%) 49 (11%) 83 (19%) 279 (63%)	6 (3.6%) 15 (9%) 36 (22%) 109 (66%)
Ethnicity - Hispanic	23%	20%	92 (21%)	29 (17%)
Comorbidities in year prior to COVID-19 hospital admission - Cancer - Cardiac arrhythmia - Chronic kidney disease - Chronic pulmonary disease - Dementia - Depression - Diabetes - Hypertension - Hypothyroidism - Obesity - Stroke/neurologic disorders	5.9% 5.6% 14% 9.0% 2.8% 7.8% 19% 2.4% 8.9% 15% 3.7%	6.3% 4.9% 18% 10% 2.5% 5.7% 21% 4.2% 9.9% 15% 5.4%	25 (5.7%) 19 (4.3%) 21 (4.8%) 37 (8.4%) 8 (1.8%) 24 (5.4%) 64 (15%) 10 (2.3%) 35 (7.9%) 60 (14%) 13 (2.9%)	11 (6.6%) 11 (6.6%) 47 (28%) 18 (11%) 4 (2.4%) 8 (4.8%) 45 (27%) 9 (5.4%) 20 (12%) 25 (15%) 13 (7.8%)
Admitted to ICU first day of hospitalization	5.9%	6.8%	19 (4.3%)	15 (9%)
CKD stage based on eGFR median value over prior year - Stage 3a - Stage 3b - Stage 4 - Stage 5	51% 7.7% 4.1% 0 (0%)	47% 8.8% 9.2% 0 (0%)	25 (5.7%) 3 (0.68%) 1 (0.23%) 0 (0%)	18 (11%) 25 (15%) 20 (12%) 0 (0%)
First day of anticoagulation in ICU	14%	16%	56 (13%)	32 (19%)
Admission diagnosis - Bacterial pneumonia - Cardiac arrhythmias - Delirium / Encephalopathy - Sepsis - Stroke	0.34% 0.99% 2.1% 4.8% 0.40%	0% 1.8% 2.1% 5.9% 2.1%	2 (0.45%) 5 (1.1%) 10 (2.3%) 18 (4.1%) 1 (0.23%)	0 (0%) 4 (2.4%) 4 (2.4%) 12 (7.2%) 4 (2.4%)
Oxygenation on day of admission - Invasive mechanical ventilation - Non-invasive mechanical ventilation - High-flow oxygen therapy - Low-flow oxygen therapy - Other form of oxygen therapy - No oxygenation	1.6% 3.1% 7.1% 36% 0.44% 59%	5.8% 2.5% 6.3% 34% 0.40% 57%	3 (0.68%) 12 (2.7%) 33 (7.5%) 191 (43%) 2 (0.45%) 233 (53%)	15 (9%) 5 (3%) 8 (4.8%) 49 (30%) 1 (0.6%) 96 (58%)
Propensity score for Heparin vs. Enoxaparin treatment (standard deviation)	0.445 (0.185)	0.522 (0.222)	0.343 (0.184)	0.658 (0.27)

**Table 7:** Summary of clinical outcomes for **weighted** cohorts of hospitalized COVID-19 patients who have taken either Heparin or Enoxaparin. For categorical variables such as mortality status and complications, patient counts are shown with the percentage of each cohort in parentheses. For numeric variables such as hospital and ICU length of stay, the mean value for each cohort is shown with standard deviation in parentheses. In addition, Benjamini-Hochberg adjusted p-values are shown for the statistical tests comparing the outcome variables for the matched Enoxaparin and Heparin cohorts.

Outcome variable	Enoxaparin, but not Heparin (Matched)	Heparin, but not Enoxaparin (Matched)	Bootstrap-based *p*-value	BH-adjusted *p*-value	Risk ratio, Heparin to Enoxaparin (95% CI)
Number of patients	441	166			
Deceased (ever)	2.7%	14%	1.1e−9	5.5e−9	5.2 (2.5, 10.7)
Deceased 28 days within first day of hospitalization (out of patients with known mortality status)	3.7%	16%	8.5e−4	2.1e−3	4.3 (1.8, 10.2)
ICU admission	24%	26%	0.69	0.69	0.74 (0.49, 1.1)
Hospital length of stay in days	5.7 (4.4)	6.5 (6.1)	0.14	0.17	
ICU length of stay in days	1.1 (2.6)	1.7 (4.7)	0.13	0.17

**Table 8:** Summary of occurrences of complications during hospitalization (days 0 to 28) for **weighted** cohorts of hospitalized COVID-19 patients who have taken either Heparin or Enoxaparin.

Complication	Enoxaparin, but not Heparin (Original)	Heparin, but not Enoxaparin (Original)	Bootstrap-based *p*-value	BH-adjusted *p*-value
Number of patients	441	166		
Bacterial pneumonia	0.61%	1.8%	1	1
Cardiac arrest	0%	0%	1	1
Cardiac arrhythmia	0.90%	0.89%	1	1
Co- or secondary infection	0.75%	4.6%	0.01	0.12
Liver dysfunction	1.03%	1.3%	1	1
Pleural Effusions	0%	0%	1	1
Pulmonary embolism	0.56%	1.6%	1	1
Stroke / Cerebrovascular incidents	0.86%	0.81%	1	1
Viral pneumonitis	15%	11%	0.19	1

### 3.3 Beyond anticoagulant type, older age and dementia are associated with higher mortality rates

The results from the Cox proportional hazard model analysis are shown in **Supplementary Table S4**. Here, we observe that the most statistically significant clinical covariate is Age (in year), with a *p*-value of 7.7e−5. Next, we observe that Enoxaparin is associated with significantly lower rates of mortality compared to unfractionated Heparin, with a hazard ratio of 0.37 (95% CI: [0.18, 0.78], *p*-value: 8.3e−3). Beyond these two covariates, the only statistically significant clinical covariate is dementia, with a hazard ratio of 3.49 (95% CI: [1.11, 11.01], *p*-value: 0.03). This analysis demonstrates that for the mortality outcome variable, anticoagulant type is the most statistically significant predictive variable in this dataset, with the exception of the Age (in years) covariate.

Both prophylactic and therapeutic doses of Enoxaparin are associated with lower rates of mortality. In Table 9, we present the mortality rates of original and matched Enoxaparin and unfractionated Heparin cohorts stratified by prophylactic vs. therapeutic dose. Among the matched cohorts, 67/96 [70%] of patients have exclusively received prophylactic doses of unfractionated Heparin, while 85/96 [89%] of patients have exclusively received prophylactic doses of Enoxaparin. Among the patients who have exclusively taken prophylactic doses with 28-day mortality outcomes available, we observe that unfractionated Heparin patients have a similar mortality rates compared to Enoxaparin patients (unfractionated Heparin: 5/39 [13%] vs. Enoxaparin: 6/49 [12%]). Among the patients who have taken either therapeutic doses or both therapeutic/prophylactic doses with 28-day mortality outcomes available, we observe that unfractionated Heparin patients have a much higher mortality rates compared to Enoxaparin patients (unfractionated Heparin: 5/22 [23%] vs. Enoxaparin: 0/6 [0%]). These results suggest that the treatment effect may depend upon the anticoagulant dose given.

**Table 9:** **Mortality rates for anticoagulants stratified by therapeutic vs. prophylactic dose.** 28-day mortality status information for original and unmatched cohorts of patients who have taken unfractionated Heparin or Enoxaparin, stratified by therapeutic vs. prophylactic dose. For each anticoagulant cohort (original and matched), mortality status information is reported for two subpopulations: **(1) Prophylactic use only:** Patients who have taken the anticoagulant at prophylactic doses exclusively, **(2) Therapeutic and Prophylactic + Therapeutic use:** Patients who have taken the anticoagulant at therapeutic doses exclusively or both prophylactic + therapeutic doses.

	Cohort	Total number of patients	Patients with 28 day mortality outcomes reported	Patients deceased at 28 days
**Original**	Unfractionated Heparin - Prophylactic Use Only	109	72	9 (13%)
	Unfractionated Heparin - Therapeutic and Prophylactic + Therapeutic Use	57	46	11 (24%)
	Enoxaparin - Prophylactic Use Only	398	216	6 (3%)
	Enoxaparin - Therapeutic and Prophylactic + Therapeutic Use	43	28	3 (11%)
**Matched**	Unfractionated Heparin - Prophylactic Use Only	67	39	5 (13%)
	Unfractionated Heparin - Therapeutic and Prophylactic + Therapeutic Use	29	22	5 (23%)
	Enoxaparin - Prophylactic Use Only	85	49	6 (12%)
	Enoxaparin - Therapeutic and Prophylactic + Therapeutic Use	11	6	0 (0%)

In **Supplementary Table S4**, we provide some additional information on the activated partial thromboplastin times (aPTT) values for the cohort of patients with therapeutic doses of unfractionated Heparin and 2+ aPTT values available. We observe that most of the patients in this sample have at least one aPTT measurement below 46 s, which we consider to be lower bound for the normal range [25]. This suggests that among patients with aPTT data available, aPTT values may have been used to guide therapeutic dosing for unfractionated Heparin.

### 3.4 Augmented curation of EHR patient notes shows that patients that were administered enoxaparin have lower rates of bleeding

We aimed to see if there were differences in occurrence of thrombotic events and bleeding amongst patients treated strictly with Heparin and those treated strictly with Enoxaparin. To do so, we used a BERT-based neural network to extract thrombotic events from EHR notes and classify the sentences sentiment [2]. If the note was dated within the timeframe of up to 28-days after that patient's first hospitalization date and contained positive sentiment for a thrombotic event or bleeding phenotype (with >= 0.9 confidence), a patient was considered to have experienced that phenotype. Similarly, we identified patients that had thrombotic events or bleeding phenotypes in the past year leading up to their hospitalization date. Thrombotic event phenotypes included: disseminated intravascular coagulation, myocardial infarction, pulmonary embolism, stroke / cerebrovascular incident, and venous thromboembolism / deep vein thrombosis. Bleeding phenotypes included: bleeding (general), hematemesis, hematoma, and purpura.

In Table 10, we present the rates of thrombotic events and bleeding during the study period (days 0 to 28) for the matched and original cohorts. We observe that the rates of thrombotic events are relatively similar between the matched cohorts. However, the incidence of bleeding phenotypes is more prevalent in the matched unfractionated Heparin cohort (unfractionated Heparin: 10/96 (6%), Enoxaparin: 1/96 (1%), *p*-value: 0.005). In Table 11, we present the rates for the matched and original cohorts for the one year prior to the study period (days −365 to −1). Here, we observe that the incidence of bleeding is slightly higher in the matched unfractionated Heparin cohort compared to the matched Enoxaparin cohort, however the results are not statistically significant (unfractionated Heparin: 14/96 (8%), Enoxaparin: 6/96 (4%), *p*-value: 0.058). The other thrombotic phenotypes are relatively similar between the two matched cohorts.

**Table 10:** **Comparison of thrombotic and bleeding events in the clinical notes from Day 0 to Day 28.** Counts of patients with thrombotic and bleeding events reported in the clinical notes in days 0 to 28 relative to the first positive PCR test among the original and matched cohorts. Presence of phenotypes in the clinical notes was determined via a BERT-based neural network model. The thrombotic and bleeding phenotypes considered included: bleeding, disseminated intravascular coagulation, hematemesis, hematoma, myocardial infarction, pulmonary embolism, purpura, stroke / cerebrovascular incident, and venous thromboembolism / deep vein thrombosis.

Phenotype	Unfractionated Heparin (Matched)	Enoxaparin (Matched)	Unfractionated Heparin (Original)	Enoxaparin (Original)
Bleeding	10 (6%)	1 (1%)	23 (14%)	10 (6%)
Disseminated intravascular coagulation	1 (1%)	0 (0%)	2 (1%)	0 (0%)
Hematemesis	0 (0%)	0 (0%)	1 (1%)	1 (1%)
Hematoma	3 (2%)	2 (1%)	17 (10%)	3 (2%)
Myocardial infarction	5 (3%)	2 (1%)	7 (4%)	4 (2%)
Pulmonary embolism	5 (3%)	3 (2%)	8 (5%)	9 (5%)
Purpura	3 (2%)	1 (1%)	3 (2%)	3 (2%)
Stroke / Cerebrovascular incident	2 (1%)	1 (1%)	5 (3%)	4 (2%)
Venous thromboembolism / deep vein thrombosis	4 (2%)	3 (2%)	7 (4%)	6 (4%)
Total patients	96	96	441	166

**Table 11:** **Comparison of thrombotic and bleeding events in the clinical notes from Day −365 to Day −1.** Counts of patients with thrombotic and bleeding events reported in the clinical notes in days −365 to −1 relative to the first positive PCR test among the original and matched cohorts. Presence of phenotypes in the clinical notes was determined via a BERT-based neural network model. The thrombotic and bleeding phenotypes considered included: bleeding, disseminated intravascular coagulation, hematemesis, hematoma, myocardial infarction, pulmonary embolism, purpura, stroke / cerebrovascular incident, and venous thromboembolism / deep vein thrombosis.

Phenotype	Unfractionated Heparin (Matched)	Enoxaparin (Matched)	Unfractionated Heparin (Original)	Enoxaparin (Original)
Bleeding	14 (8%)	6 (4%)	31 (19%)	40 (24%)
Disseminated intravascular coagulation	0 (0%)	0 (0%)	0 (0%)	0 (0%)
Hematemesis	0 (0%)	0 (0%)	0 (0%)	1 (1%)
Hematoma	5 (3%)	2 (1%)	19 (11%)	14 (8%)
Myocardial infarction	4 (2%)	4 (2%)	9 (5%)	8 (5%)
Pulmonary embolism	1 (1%)	1 (1%)	3 (2%)	6 (4%)
Purpura	3 (2%)	2 (1%)	6 (4%)	7 (4%)
Stroke / Cerebrovascular incident	1 (1%)	2 (1%)	6 (4%)	9 (5%)
Venous thromboembolism / deep vein thrombosis	3 (2%)	2 (1%)	10 (6%)	9 (5%)
Total patients	96	96	441	166

## 4 Discussion

This retrospective study highlights interesting differences in the outcomes associated with the administration of Enoxaparin vs. unfractionated Heparin in COVID-19 patients. We find that Enoxaparin is associated with lower 28-day mortality compared to unfractionated Heparin, even after controlling for potential confounding factors such as demographics, comorbidities, admission diagnosis, initial ICU status, and initial level of oxygen support. We observe that these differences are primarily driven by differences in the mortality rates among patients who received therapeutic doses of anticoagulants. Furthermore, we find that patients administered Enoxaparin experienced lower rates of bleeding events during the 28-day study period compared to patients administered unfractionated Heparin.

Previous studies have suggested that Enoxaparin may be more effective than unfractionated Heparin in certain cases for the treatment and prophylaxis of coagulopathies. A meta-analysis of four clinical trials with 3600 patients in total was conducted to evaluate the relative efficacy and safety of Enoxaparin and unfractionated Heparin for the prevention of VTE in hospitalized patients [26]. This meta-analysis found that the Enoxaparin cohort had significantly reduced rates of VTE and all-cause mortality compared to the unfractionated Heparin cohort without increased rates of major bleeding [26]. A large multi-hospital study involving over 3000 patients found that patients receiving Enoxaparin had a 74% lower risk of VTE compared to patients receiving unfractionated Heparin prophylactically [13]. In another meta-analysis comparing Enoxaparin and unfractionated Heparin, Enoxaparin was associated with higher efficacy as adjunctive antithrombin therapy among over 49,000 patients across the acute coronary syndromes spectrum [27]. In our study, we also find that the Enoxaparin cohort had favorable outcomes compared to the unfractionated Heparin cohort, in the setting of prophylactic and therapeutic anticoagulant treatments for COVID-19.

There are a few limitations of this study. First, Enoxaparin and Heparin differ in FDA label indications. The label for Enoxaparin [24] includes the prophylaxis and treatment of deep vein thrombosis (DVT) with or without pulmonary embolism (PE) in various settings, the prophylaxis of ischemic complications of unstable angina and non-Q-wave myocardial infarction (MI), and the treatment of acute ST-segment elevation MI managed medically or with subsequent percutaneous coronary intervention. On the other hand, the label for unfractionated Heparin [28] includes similar prophylactic indications as well as the treatment of a broader spectrum of acute embolic events including peripheral arterial embolism and embolism in the setting of atrial fibrillation, the treatment of consumptive coagulopathies, and usage as an anticoagulant in high-risk patient groups such as those undergoing blood transfusions, extracorporeal circulation, and dialysis procedures. As a result, it is possible that the patient population receiving unfractionated Heparin is more severely or acutely ill to begin with.

Another limitation of this study is that the data were not created or collected to answer the specific research question for this analysis. As a result, the results of this study may be influenced by unmeasured confounding variables which are not recorded in this dataset, such as socioeconomic factors. In addition, there may be misclassification bias if the anticoagulant medications for some of these patients were entered incorrectly into the EHR. Due to the relatively short time period of this study, bias due to changing eligibility criteria over time is unlikely because the medication codes to identify the cohorts of patients who were administered Enoxaparin and unfractionated Heparin has remained constant over the course of the study.

Overall, the results of this study motivate future studies to investigate biological mechanisms underlying differences in the outcomes and future trials that could enable the development of more efficacious standard of practice in regards to administration of anticoagulants in COVID-19 patients. Prospective analyses comparing the efficacy of Enoxaparin and unfractionated Heparin are warranted. In follow-up analyses, it may be interesting to consider outcomes stratified by initial laboratory values such as eGFR and d-dimer levels as well.

## Data sharing statement

After publication, the data will be made available to others upon reasonable requests to the corresponding author. A proposal with detailed description of study objectives and statistical analysis plan will be needed for evaluation of the reasonability of requests. Deidentified data will be provided after approval from the corresponding author and Mayo Clinic. Supplemental information such as the programming code for this study may be made available following the same procedure for data requests.

## Authors’ contributions

Conceptualisation: CP, AV, CK, VS. Data Curation: CP, AV, CK, GB. Formal analysis: CP, AV, CK, AP. Investigation: CP, AV, CK, GB, AP. Methodology: CP, AP. Project administration: CP, AV. Resources: JO, AB, VS. Software: CP, CK, GB, AP. Supervision: VS. Writing - reviewing and editing: all authors.

## Declaration of Interests

CP, AJV, CK, GB, AP, and VS have financial interests in Nference, Inc. ADB is supported by grants from NIAID (grants AI110173 and AI120698) Amfar (#109593) and Mayo Clinic (HH Shieck Khalifa Bib Zayed Al-Nahyan Named Professorship of Infectious Diseases). ADB is a paid consultant for Abbvie and Flambeau Diagnostics, is a paid member of the DSMB for Corvus Pharmaceuticals and Equilium, owns equity for scientific advisory work in Zentalis and Nference, and is founder and President of Splissen therapeutics. JCO reports personal fees from Elsevier, Inc, personal fees from Bates College, and grants from Nference, Inc, outside the submitted work. CP, AJV, CK, GB, AP, and VS are nference employees. The Mayo Clinic has a Financial Conflict of Interest in technology used in the research and may stand to gain financially from the successful outcome of the research. This research has been reviewed by the Mayo Clinic Conflict of Interest Review Board and is being conducted in compliance with Mayo Clinic Conflict of Interest policies.

## Appendix Supplementary materials

Image, application 1

Image, application 2

Image, application 3

Image, application 4

Image, application 5
