# Supplementary material for: Enoxaparin is associated with lower rates of mortality than unfractionated Heparin in hospitalized COVID-19 patients
Source: eClinicalMedicine. 2021 Mar 9;33:100774. doi: 10.1016/j.eclinm.2021.100774 (PMC7941023; doi:10.1016/j.eclinm.2021.100774)
Supplement: Supplementary file 1 [file mmc1.docx]

**Supplementary Table S1: Anticoagulant agents used to define the Enoxaparin and unfractionated Heparin patient cohorts.** For each anticoagulant agent, we list the total number of patients in the study population which have received that anticoagulant agent as recorded in the Mayo Clinic EHR. We provide the anticoagulant agents used to define the following cohorts: **(A) Unfractionated heparin** and **(B) Enoxaparin**.

1. **Anticoagulant agents used to define the Unfractionated Heparin cohort.**

| **Medication name** | **Patient Count** |
| --- | --- |
| HEPARIN (PORCINE) 5,000 UNITS/ML INJECTION SOLUTION (WRAPPER) | 142 |
| HEPARIN (PORCINE) 1,000 UNIT/ML INJECTION SOLUTION | 54 |
| HEPARIN (PORCINE) 25,000 UNIT/250 ML (100 UNIT/ML) IN DEXTROSE 5 % IV | 48 |
| HEPARIN (PORCINE) 25,000 UNIT/250 ML IN 0.45 % SODIUM CHLORIDE IV SOLN | 13 |
| HEPARIN LOCK FLUSH (PORCINE) 100 UNITS/ML IV (WRAPPER) | 11 |
| HEPARIN (PORCINE) 2,500 UNIT/500 ML (5 UNIT/ML) IN 0.9 % NACL IV SOLN | 5 |
| HEPARIN (PORCINE) 2,000 UNIT/1,000 ML IN 0.9% NACL (WRAPPER FOR ARZ) | 2 |
| HEPARIN (PORCINE) 5,000 UNIT/500 ML (10 UNIT/ML) IN 0.9 % NACL FLUSH SOLUTION | 2 |
| HEPARIN, PORCINE (PF) 10 UNITS/ML INTRAVENOUS SOLUTION (WRAPPER) | 2 |
| HEPARIN (PORCINE) (PF) 1,000 UNIT/500 ML IN 0.9 % SODIUM CHLORIDE IV | 1 |
| HEPARIN (PORCINE) 2,000 UNIT/1,000 ML IN 0.9% NACL (WRAPPER) FLUSH INFUSION | 1 |
| HEPARIN, PORCINE (PF) 1 UNIT/ML INTRAVENOUS SYRINGE | 1 |
| HEPARIN, PORCINE (PF) 5,000 UNIT/0.5 ML INJECTION (WRAPPER) | 1 |

1. **Anticoagulant agents used to define the Enoxaparin cohort.**

| **Medication name** | **Patient Count** |
| --- | --- |
| ENOXAPARIN 40 MG/0.4 ML SUBCUTANEOUS SYRINGE | 425 |
| ENOXAPARIN 30 MG/0.3 ML SUBCUTANEOUS SYRINGE | 36 |
| ENOXAPARIN 80 MG/0.8 ML SUBCUTANEOUS SYRINGE | 18 |
| ENOXAPARIN 100 MG/ML SUBCUTANEOUS SYRINGE | 10 |
| ENOXAPARIN 60 MG/0.6 ML SUBCUTANEOUS SYRINGE | 7 |
| ENOXAPARIN 150 MG/ML SUBCUTANEOUS SYRINGE | 2 |
| ENOXAPARIN 120 MG/0.8 ML SUBCUTANEOUS SYRINGE | 1 |
| ENOXAPARIN 300 MG/3 ML SUBCUTANEOUS SOLUTION | 1 |
