# Supplementary material for: Enoxaparin is associated with lower rates of mortality than unfractionated Heparin in hospitalized COVID-19 patients
Source: eClinicalMedicine. 2021 Mar 9;33:100774. doi: 10.1016/j.eclinm.2021.100774 (PMC7941023; doi:10.1016/j.eclinm.2021.100774)
Supplement: Supplementary file 2 [file mmc2.docx]

**Supplementary Table S2: Diagnosis Code Definitions.** ICD-10 codes used to define the clinical covariates for the study population, including: (A) Complications, (B) Admission Diagnoses, and (C) Comorbidities.

1. **ICD-10 codes used to define complications for the study population.**

| **Complications** | **ICD-10** |
| --- | --- |
| Acute cardiac injury | S26 |
| Acute kidney injury | N17 |
| Anemia | D64.9;D64.8 |
| Bacteremia | R78.81 |
| Bacterial pneumonia | J15 |
| Cardiac arrest | I46 |
| Cardiac arrhythmia | I49 |
| Co- or secondary infection | A06.89;A69;B00.89;B00.9;N46.122;B08.6;D70.3;K94.12;K94.32;N99.511;A60.01;O23.2;O23.51;A02.29;A06.8;B00.8;O41.1;O75.3;P35-P39;B08.0;B34.4;O86.0;A54.29;A60.9;H59.4;A50-A64;K95.81;M46.3;A31;N98.0;K94.22;O99.830;O99.835;Z22.4;R65;O98.3;O99.83;Z20.2 |
| Congestive heart failure | I50.2;I50.3;I50.4 |
| Deep vein thrombosis | I82.49;I82.59 |
| Hyperglycemia | R73.9 |
| Liver dysfunction | K70-K77 |
| Pleural Effusion | J90;J91 |
| ARDS | J80;R06.03 |
| Septic shock | R65.21 |
| Stroke / Cerebrovascular incidents | I60-I69 |
| Viral pneumonitis | J12 |

1. **ICD-10 codes used to define admission diagnoses for the study population.**

| **Admission Diagnoses** | **ICD-10** |
| --- | --- |
| Acute hypoxic respiratory failure | J96.01 |
| Acute kidney injury | N17 |
| ARDS | J80;R06.03 |
| Bacterial pneumonia | J15 |
| Cardiac arrest | I46 |
| Cardiac arrhythmias | I49 |
| Congestive heart failure | I50.2;I50.3;I50.4 |
| Delirium / Encephalopathy | R41;G93.4 |
| Hyperglycemia | R73.9 |
| Sepsis | A41;R65.2 |
| Shock | R57.1 |
| Stroke | I60-I69 |

1. **ICD-10 codes used to define comorbidities for the study population.**

| **Comorbidities** | **ICD10** |
| --- | --- |
| Asthma | J45 |
| Cancer | C69.01;C69.02;C69.20;C69.21;C69.22;C69.61;C69.62;C72.1;C75.2;C00.8;C30-C39;R53.0;Z80;C12;O9A.11;O9A.12;O9A.13;C00.2;C10.4;C51-C58;C68.9;C76.40;C76.41;C76.42;C76.50;C76.51;C76.52;C80;C75.8;C06;C45-C49;C63;C68;C76;C76-C80;C81-C96;Z08;Z09 |
| Cardiac arrhythmia | I49 |
| Chronic dialysis | Z99.2 |
| Chronic kidney disease | N18 |
| Chronic pulmonary disease | J40-J47 |
| Congestive heart failure | I50.2;I50.3;I50.4 |
| Coronary artery disease | I25.1;I25.75;I25.7;I25.810;I25.811;I25.812 |
| Dementia | R41.81;F03 |
| Depression | F33 |
| Diabetes | E08-E13 |
| Hypertension | I10;I11 |
| Hypothyroidism | E02;E03 |
| Obesity | E66 |
| Obstructive sleep apnea | G47.33 |
| Stroke/neurologic disorder | I60-I69 |
