# Supplementary material for: Enoxaparin is associated with lower rates of mortality than unfractionated Heparin in hospitalized COVID-19 patients
Source: eClinicalMedicine. 2021 Mar 9;33:100774. doi: 10.1016/j.eclinm.2021.100774 (PMC7941023; doi:10.1016/j.eclinm.2021.100774)
Supplement: Supplementary file 3 [file mmc3.docx]

**Supplementary Table S3:** Cause of death information for matched cohorts of patients administered Enoxaparin only or Heparin only. The cause of death information was determined by a manual review of the clinical notes for each of the 8 deceased patients in the matched Enoxaparin cohort and for each of the 21 deceased patients in the matched Heparin cohort. Each row corresponds to a patient, with fields indicating whether the patient had one of the following conditions: ARDS, AKI, Sepsis, Pneumonia, Heart Failure, or Other (any other medical condition). In addition, the last column indicates if the cause of death could not be determined for the particular patient.

| **Index** | **Anticoagulant** | **ARDS / Acute respiratory failure / Hypoxia** | **AKI / Renal Failure** | **Sepsis** | **Pneumonia** | **Heart Failure** | **Other** | **Inconclusive** |
| --- | --- | --- | --- | --- | --- | --- | --- | --- |
| 1 | Enoxaparin | yes |  |  | yes |  |  |  |
| 2 | Enoxaparin |  | yes |  |  |  | yes |  |
| 3 | Enoxaparin |  | yes |  |  |  | yes |  |
| 4 | Enoxaparin | yes |  |  | yes |  |  |  |
| 5 | Enoxaparin |  |  |  | yes |  | yes |  |
| 6 | Enoxaparin |  |  |  |  |  | yes |  |
| 7 | Enoxaparin | yes | yes |  |  | yes | yes |  |
| 8 | Enoxaparin | yes |  |  | yes |  |  |  |
| 1 | Heparin | yes | yes | yes | yes |  |  |  |
| 2 | Heparin | yes | yes |  |  | yes |  |  |
| 3 | Heparin |  |  |  |  |  |  | yes |
| 4 | Heparin |  |  |  |  |  |  | yes |
| 5 | Heparin | yes |  | yes | yes |  |  |  |
| 6 | Heparin | yes |  | yes | yes |  |  |  |
| 7 | Heparin | yes | yes |  | yes |  |  |  |
| 8 | Heparin |  |  |  |  |  |  | yes |
| 9 | Heparin | yes |  | yes | yes | yes |  |  |
| 10 | Heparin | yes | yes |  | yes |  |  |  |
| 11 | Heparin | yes |  |  | yes | yes |  |  |
| 12 | Heparin | yes | yes |  | yes |  |  |  |
| 13 | Heparin | yes |  |  | yes |  |  |  |
| 14 | Heparin | yes |  |  | yes |  |  |  |
| 15 | Heparin | yes |  | yes | yes |  |  |  |
| 16 | Heparin | yes |  |  | yes | yes |  |  |
| 17 | Heparin |  |  |  |  |  |  | yes |
| 18 | Heparin |  |  |  |  |  |  | yes |
| 19 | Heparin |  | yes |  |  | yes |  |  |
| 20 | Heparin | yes |  |  | yes |  |  |  |
| 21 | Heparin | yes |  |  | yes |  |  |  |
