# Supplementary material for: Enoxaparin is associated with lower rates of mortality than unfractionated Heparin in hospitalized COVID-19 patients
Source: eClinicalMedicine. 2021 Mar 9;33:100774. doi: 10.1016/j.eclinm.2021.100774 (PMC7941023; doi:10.1016/j.eclinm.2021.100774)
Supplement: Supplementary file 4 [file mmc4.docx]

**Supplementary Table S4: Cox proportional hazard model output.** Hazard ratios for mortality generated by a Cox proportional hazard model, along with 95% confidence intervals and p-values. For each categorical covariate, the hazard ratio is relative to a reference value for that covariate, which is specified in the table. For each numeric covariate, the hazard ratio is relative to a single unit increase for that covariate, according to the units specified in the table. To indicate statistical significance, * denotes a p-value between 0.05 and 0.01, ** denotes a p-value between 0.01 and 0.001, and *** denotes a p-value less than 0.001.

| **Covariate** | **Hazard ratio (95% CI)** | **p-value** |
| --- | --- | --- |
| Anticoagulant (reference = Unfractionated Heparin)   - Enoxaparin | 0.37 [0.18, 0.78] | 8.30E-03** |
| Age (in years) | 1.05 [1.02, 1.07] | 7.70E-05*** |
| Gender (reference = Female)   - Male | 1.38 [0.69, 2.78] | 0.37 |
| Race (reference = White)   - Asian - Black - Other | 0.59 [0.11, 3.10]  0.40 [0.09, 1.72]  1.26 [0.52, 3.07] | 0.54  0.22  0.61 |
| Ethnicity (reference = Non-Hispanic)   - Hispanic - Other | 0.57 [0.20, 1.63]  0.78 [0.12, 5.01] | 0.29  0.79 |
| Oxygenation status on hospital admission  (reference = none)   - Invasive mechanical ventilation - Low-flow oxygen - Non-invasive mechanical ventilation - Other | 2.07 [0.65, 6.58]  1.01 [0.51, 1.99]  0.86 [0.13, 5.89]  0.39 [0.0, 1398]- | 0.22  0.99  0.88  0.82 |
| Chronic Kidney Disease (reference = none)   - Stage 3a (eGFR: 45-59%) - Stage 3b (eGFR: 30-44%) - Stage 4 (eGFR: 15-29%) | 1.00 [0.37, 2.72]  0.89 [0.27, 2.88]  2.37 [0.88, 6.38] | 1.00  0.84  0.09 |
| Comorbidity   - Cancer (reference = none) - Cardiac arrhythmias (reference = none) - Chronic pulmonary disease (reference = none) - Dementia (reference = none) - Depression (reference = none) - Hypertension (reference = none) - Hypothyroidism (reference = none) - Obesity (reference = none) - Pulmonary embolism (reference = none) - Stroke or other neurologic disorders   (reference = none)   - Type 1 diabetes mellitus (reference = none) - Type 2 diabetes mellitus (reference = none) | 1.38 [0.49, 3.91]  1.52 [0.48, 4.85]  0.81 [0.29, 2.25]  3.49 [1.11, 11.01]  1.02 [0.25, 4.10]  0.60 [0.09, 3.87]  0.62 [0.21, 1.86]  0.54 [0.18, 1.62]  7.07 [0.66, 75.51]  2.47 [0.78, 7.82]  2.23 [0.25, 19.62]  1.26 [0.58, 2.7] | 0.54  0.47  0.69  0.03*  0.98  0.59  0.39  0.27  0.11  0.12  0.47  0.56 |
| Admitted to the ICU on first day of hospital admission (reference = False)   - True | 1.81 [0.55, 5.88] | 0.33 |
| Admitted to the ICU on first day of anticoagulant administration (reference = False)   - True | 1.95 [0.73, 5.20] | 0.18 |
